# Use of Dried Blood Spots to Elucidate Full-Length Transmitted/Founder HIV-1 Genomes

**DOI:** 10.20411/pai.v1i1.116

**Published:** 2016-07-20

**Authors:** Jesus F. Salazar-Gonzalez, Maria G. Salazar, Damien C. Tully, Colin B. Ogilvie, Gerald H. Learn, Todd M. Allen, Sonya L. Heath, Paul Goepfert, Katharine J. Bar

**Affiliations:** 1 Comprehensive Cancer Center, University of Alabama at Birmingham, Birmingham, Alabama; 2 Department of Medicine, University of Alabama at Birmingham, Birmingham, Alabama; 3 Ragon Institute of MHG, MIT, and Harvard, Boston, Massachusetts; 4 Department of Medicine, Perelman School of Medicine, University of Pennsylvania, Philadelphia, Pennsylvania

**Keywords:** dried blood spots, single genome sequencing, transmitted/founder virus, viral diversity, resource-limited settings

## Abstract

**Background:**

Identification of HIV-1 genomes responsible for establishing clinical infection in newly infected individuals is fundamental to prevention and pathogenesis research. Processing, storage, and transportation of the clinical samples required to perform these virologic assays in resource-limited settings requires challenging venipuncture and cold chain logistics. Here, we validate the use of dried-blood spots (DBS) as a simple and convenient alternative to collecting and storing frozen plasma.

**Methods:**

We performed parallel nucleic acid extraction, single genome amplification (SGA), next generation sequencing (NGS), and phylogenetic analyses on plasma and DBS.

**Results:**

We demonstrated the capacity to extract viral RNA from DBS and perform SGA to infer the complete nucleotide sequence of the transmitted/founder (TF) HIV-1 envelope gene and full-length genome in two acutely infected individuals. Using both SGA and NGS methodologies, we showed that sequences generated from DBS and plasma display comparable phylogenetic patterns in both acute and chronic infection. SGA was successful on samples with a range of plasma viremia, including samples as low as 1,700 copies/ml and an estimated ~50 viral copies per blood spot. Further, we demonstrated reproducible efficiency in gp160 *env* sequencing in DBS stored at ambient temperature for up to three weeks or at -20°C for up to five months.

**Conclusions:**

These findings support the use of DBS as a practical and cost-effective alternative to frozen plasma for clinical trials and translational research conducted in resource-limited settings.

## INTRODUCTION

Accurate identification and characterization of the virus that establishes productive clinical HIV-1 infection, or the transmitted/founder (TF) virus, is fundamentally important to many areas of HIV research. The exact identification of TF HIV-1 genomes was first enabled by single genome amplification strategies (SGA) [1–7], which utilize endpoint dilution amplification of plasma viral HIV-1 RNA to accurately and proportionally represent full-length genes or genomes from the complex circulating virus quasispecies. SGA has been an enabling technology for TF identification because it allows linkage between distant sites by sequencing of longer continuous templates and minimizes *in vitro* errors associated with bulk polymerase chain reaction (PCR) techniques [4, 8]. SGA applied to acutely HIV-1 infected cohorts enabled the enumeration and characterization of TF viruses and demonstrated the strict transmission bottleneck existent in heterosexual, men who have sex with men (MSM), and injection drug user (IDU) transmission [1–5, 9], and the immune pressures at play in acutely infected individuals [7, 10, 11]. More recently, identification of the TF viruses that break through in clinical vaccine trials has enabled genetic and phenotypic sieve studies [9, 12–17]. By comparing features of the TF viruses establishing infection in vaccine recipients to those of placebo recipients, sieve analyses have identified properties of the specific viruses blocked (or “sieved”) by vaccine-induced immune pressure. Examples include CD8 T-cell epitopes in conserved viral structural proteins [15] and antibody targets in envelope [14]. In addition, SGA analysis of the breakthrough rebound viruses that arise after or during clinical trials of curative strategies [18, 19] has the potential to elucidate the mechanisms of successful virus suppression or eradication and the pathways that viruses utilize to escape. For example, SGA and phenotypic characterization of breakthrough viruses emerging during or after passive administration of broadly neutralizing antibodies (bNAbs) allows accurate identification of neutralization escape variants and their pathways of developing resistance [20]. The HIV-1 field is currently testing multiple novel prevention strategies in large clinical trials, including vaccines, passively administered bNAbs, pre-exposure prophylaxis, and microbicides. For each of these trials, identifying and analyzing the breakthrough TF viruses for relevant properties (*e.g.,* drug resistance, neutralization sensitivity, linked transmission networks, etc.) will be of paramount importance to the interpretation of trial results.

Accurate identification of TF or breakthrough viruses requires frequent venipuncture to obtain plasma samples near the time of transmission or rebound and places significant burdens on trial subjects and clinical staff (repeated venipuncture) and infrastructure (cold storage and shipping of many plasma samples). For these critically important trials, especially those conducted in resource-limited settings, simple and cost-effective protocols are needed. The capacity to use dried blood spots (DBS) to identify and study the breakthrough TF viruses, in lieu of stored plasma samples, would greatly facilitate cost-effective clinical trials.

The past several years have yielded multiple demonstrations of the feasibility of DBS as an alternative clinical specimen for HIV-1 diagnosis, viral load quantification, and drug-resistance geno-typing [21–27]. The World Health Organization has identified DBS as the sample type for HIV resistance genotyping in settings without the capacity to collect, store, and transport plasma [28]. DBS have important advantages over plasma, particularly in resource-limited settings, as they are simple to collect, represent non-hazardous material, and require substantially less strict cold storage, in terms of both space and temperature for storage and transportation [21]. While studies of paired DBS and plasma samples have reported concordant results for HIV-1 genotyping and viral load quantification [21, 22, 26, 27], no previous study has demonstrated the feasibility of using DBS to examine HIV-1 *envelope (env*) gene diversity in acute and chronic infection or identify the TF *env* or full-length genome during primary HIV-1 infection. Recently, investigators presented an in-house SGA assay to amplify HIV-1 DNA from DBS obtained from infant heel-prick blood in field settings in Lusaka, Zambia [29], but no D Lusaka, Zambia NA sequencing data were included. Here, we examined the feasibility of using DBS to delineate HIV *env* diversity in acute and chronic infection and to identify the TF *envs* and complete genomes responsible for establishing productive clinical infection. We found that SGA from DBS provided equivalent sequencing results compared to those derived from plasma, and sufficient efficiency to merit its consideration in future clinical trials.

## MATERIAL AND METHODS

**Patients.** Peripheral blood samples were obtained from 9 subjects with either acute/early (n = 2) or chronic (n = 7) HIV infection. All patients provided written informed consent for blood collection and subsequent analysis, under protocols approved by the Institutional Review Board of the University of Alabama at Birmingham. Clinical and laboratory staging of HIV infection was estimated by a battery of commercial assays, including HIV-1 enzyme immunoassay, Western blot, and quantitative polymerase chain reaction.

**DBS and plasma collection.** Whole blood was collected by venipuncture from HIV-infected individuals in tubes containing acid-citrate-dextrose (ACD) as an anticoagulant. Plasma was separated within 6 hours of the time of blood collection by centrifugation, aliquoted in clean tubes, and stored at −80°C. DBS were prepared at the University of Alabama at Birmingham Center for AIDS Research laboratories by pipetting 50 μl of freshly collected ACD whole blood in each of 5 circles of a Whatman 903 card (Whatman Inc., Florham Park, NJ). Cards from each patient were then allowed to air-dry in a flat position at ambient temperature for 2-4 hours, followed by short-term storage at ambient temperature (~23°C) in a sealed plastic bag along with silica gel desiccant sachets. After short-term storage, DBS enclosed in sealed bags were transferred to a non-frost-free −20°C freezer for long-term storage.

**Nucleic acid extraction from plasma and DBS.** Individual 50 μl DBS were submitted to RNA extraction by two techniques based on DBS card manufacturer instructions. For technique 1, DBS were incubated with 400 μl of RNase-free water for 30 minutes at 37°C, followed by transfer of the reddish fluid to a clean microcentrifuge tube containing 1 ml of TRIzol reagent (Life Technologies). In technique 2, DBS were directly incubated in 1 ml of TRIzol reagent (Life Technologies) for 30 minutes at room temperature, followed by transfer of the TRIzol-sample mix to a clean microcentrifuge tube. Plasma samples were similarly analyzed in two ways. For the first method, 1 ml of TRIzol was mixed and incubated with 25 μl of liquid plasma (the estimated average amount of plasma dried in 50 μl blood spots) for 30 minutes at room temperature. At the end of the incubation, RNA was extracted from the TRIzol containing samples following the manufacturer's instructions (Life Technologies). For the second method, RNA was extracted from 25 μl of liquid plasma using a QIAamp viral RNA mini kit and following the manufacturer's instructions (QIAGEN, Valencia, CA), which served as a reference RNA standard to estimate the efficiency of SGA assays performed in all other RNA extraction procedures. Total RNA was isolated via precipitation, washed, and resuspended in 30 μl of RNase-free water (for procedures using TRIzol) or 60 μl of elution buffer (for the QIAGEN protocol), as per the manufacturers' instructions. RNA preparations were used either immediately for cDNA synthesis or stored at −80°C.

**cDNA synthesis.** Reverse transcription was carried out with SuperScript III, using the protocol recommended by the manufacturer (Invitrogen Life Technologies, Carlsbad, CA) as previously described [4–6]. Briefly, 15 μl of nucleic acid extract from either plasma or DBS was reverse-transcribed in a final volume of 40 μl of 1x reverse transcription buffer, 5 mM dithiothreitol, 0.5 mM of each deoxynucleoside triphosphate (dNTPs), 0.25 μM antisense primer, 2 units/μl RNase inhibitor, and 10 units/μl SuperScript III. Reverse primer sequences to generate cDNAs for *env*, 5′-and 3′-half genome amplification have been previously published [4, 7]. Reactions were incubated at 50°C for 60 min, followed by an additional hour at 55°C. At the end of the incubation, reactions were stopped by heat (70°C for 15 min), followed by RNaseH digestion at 37°C for 20 min. Tubes containing cDNA were then immediately used for SGA or stored at −80°C.

**Single genome amplification and sequencing.** To amplify full-length *env* genes, half-genomes and near full-length genomes (9-Kb), cDNA was endpoint diluted to an estimated single copy per well, followed by nested PCR using SGA procedures described previously [4, 6]. Primers were slightly different to amplify the 5′-half fragment for this study, as indicated below. Briefly, firstand second-round PCR were carried out similarly in 20 μl reactions containing 1x High Fidelity Platinum PCR buffer, 2 mM MgSO4, 0.2 mM of each dNTP, 0.2 μM each of sense and antisense primers, and 0.025 units/μl platinum Taq High Fidelity polymerase (Invitrogen, Carlsbad, CA) in MicroAmp optical 96-well reaction plates (Thermo Scientific, Foster City, CA) and sealed with ABI MicroAmp adhesive film. 5′-half genomes were amplified in the first-round PCR using sense primer RVF1 5′-GGG TCT CTC TGG TTA GAC CAG ATC-3′ (nt 454-477 HXB2) and antisense primer B5R1 5′-CTT GCC ACA CAA TCA TCA CCT GCC AT-3′ (nt 5,052–5,077 HXB2) using the following PCR conditions: 94°C for 2 minutes followed by 35 cycles of 94°C for 15 seconds; 55°C for 30 seconds; and 68°C for 5 minutes, with a final extension of 68°C for 10 minutes. Next, 1 or 2 μl of first-round product was transferred to a final 20 μl PCR reaction containing sense primer RVF20 5′-GAG CCT GGG AGC TCT CTG GCT A (nt 479–500 HXB2) and antisense primer B5R2 5′- CAA TCA TCA CCT GCC ATC TGT TTT CCA TA-3′ (nt 5,040–5,068 HXB2), and a ~5 Kb fragment was amplified using the same reagent mix as the first round. PCR conditions were the same as the first round, but with a total of 45 PCR cycles. PCR positive wells containing amplicons of the predicted size by agarose gel electrophoresis were selected for DNA sequencing. Sanger sequencing was performed in a 3730xl DNA Analyzer using BigDye Terminator v.3.1 chemistry following the manufacturer's protocols (Thermo Scientific, Carlsbad, CA). Amplicons were sequenced bidirectionally and chromatograms were edited using Sequencher program, version 5.1 (Gene Codes Corporation, Ann Arbor, MI). PCR products displaying mixed bases at one or multiple nucleotide positions were excluded from further analysis.

**Efficiency comparisons.** The estimated efficiency of the method to amplify HIV-1 from single molecules was based on the number of amplicons derived from a unit of plasma equivalents present in paired DBS and plasma extracts. We assigned 100% efficiency to HIV amplification from frozen plasma extracted with the QIAamp viral RNA mini kit. The efficiency in DBS was then calculated using the number of amplicons derived from plasma equivalents present in the DBS extract after considering that approximately half the volume of the 50 μl of whole blood spotted on the filter paper is plasma.

**Next generation 454 deep sequencing.** DBS vRNA from samples T8250 and N8261 were amplified using nested reverse transcriptase PCR (RT-PCR) over three overlapping regions of the genome (*gag*, *pol*, and a 3′ genomic half). Primer sequences and PCR conditions are available upon request. First-round RT-PCR was performed using the Superscript III one-step RT-PCR system with Platinum *Taq* DNA polymerase with High Fidelity (Invitrogen, Carlsbad, CA). Each RTPCR reaction was composed of 10 μl of RNA, 25 μl of 2x reaction mix (buffer, deoxynucleoside triphosphate, and MgSO_4_), 0.4 μM of forward and reverse primers and 1 μl of enzyme mix, and water. Takara Ex *Taq* DNA polymerase Hot Start enzyme (Takara Bio Inc., Shiga, Japan) was used for the second-round PCR using 2 μl of round 1 product and 0.6 μM of each primer according to the manufacturer's directions. For post-PCR quality control purposes, the products were run on precast 1% agarose E-gels. Positive PCR products were purified using the QIAquick PCR Purification Kit (Qiagen) or the PureLink Quick Gel Extraction Kit (Invitrogen) and concentrations were determined using the Thermo Scientific Nanodrop 8000 spectrophotometer (Nanodrop Products). Amplifications from the same subject were pooled with concentration ratios of *gag*: *pol*: 3′ half being 1:1:3.

Purified PCR products from each sample were pooled in equimolar ratios, fragmented using the transposon-mediated Nextera DNA Sample Prep Kit (Illumina) following the manufacturer's protocol, and purified with a DNA Clean and Concentrator Kit (Zymo Research). Adapters and barcodes were added by limited-cycle PCR (Nextera). Small fragments were removed following Roche's recommended size selection protocol and quantified using a Promega Quantiflor-ST fluorometer. After quantification, barcoded samples were pooled at a final concentration of 10^7 molecules/μl to create the library for sequencing on the GS Junior 454 Genome Sequencer (Roche Diagnostics). Emulsion PCR, breaking, and DNA sequencing were performed according to the manufacturer's protocols for Lib-L (Roche Diagnostics). Resulting sequence reads were then cleaned and assembled into a *de novo* consensus using AV454 and VICUNA [30, 31]. Reads were first clustered to form contigs using min hashes and an inexact string matching based search to infer similarity. Contigs were validated by iteratively aligning each constituent read to the consensus, and then aggressively merged to form a *de novo* consensus assembly of the viral population.

The reads were then corrected for systematic 454 errors such as homopolymer indels and carry-forward/incomplete extensions and aligned to the consensus assembly using the previously developed software RC454 [30] with a variant calling performed by V-Phaser [32]. Briefly, V-Phaser uses phase and quality filtering with a probability model that recalibrates quality scores for individual bases to iteratively refine probabilities and to define the threshold required to statistically call variants in the presence of errors with high specificity and high sensitivity [32]. Analyses were further subjected to manual inspection to identify and discard any sequencing artifacts.

**Phylogenetic analysis.** SGA-derived *env* nucleotide sequences from each subject were aligned using ClustalW, and manually adjusted [33]. Intrapatient *env* diversity was determined by pairwise comparison of sequences using uncorrected sequence distances. For each subject, a maximum likelihood (ML) phylogenetic tree was inferred using PhyML, version 3.0 [34], and phylogenetic support was based on 100 bootstrap replicates. The genetic identity of TF *env* genes in individuals with recent infection was inferred as described previously [4]. APOBEC-associated G-to-A hypermutated sequences were excluded from analysis in the inference of the TF *env* sequence according to the Los Alamos National Laboratory (LANL) HIV Sequence Database tool Hypermut 2.0 [35]: http://www.hiv.lanl.gov/content/sequence/HYPERMUT/hypermut.html. Highlighter analysis of SGA sequences was performed using the LANL Highlighter tool: http://www.hiv.lanl.gov/content/sequence/HIGHLIGHT/highlighter_top.html. Poisson-fitter analyses were performed to determine whether *env* sequences conformed to predictions of a mathematical model of random viral evolution and to estimate the time to most recent common ancestor (MRCA) or the minimum number of days required to explain the observed within-patient HIV-1 sequence diversification from a single common ancestor sequence. This analysis was done using the Poisson-Fitter tool from the LANL (http://www.hiv.lanl.gov/content/sequence/POISSON_FITTER/poisson_fitter.html), as previously described [4, 36].

**Statistical analysis.** Evidence of statistical compartmentalization was assessed using programs implemented by HyPhy software [37] including the Slatkin-Maddison (SM) test, which compares the minimum number of intercompartmental migration events to the frequency distribution of migration events in 1,000 randomized trees [38]. Two-sample tests were performed to compare intra-patient sequence diversity between DBS vs plasma viral populations using the DIVEIN web tool (http://www.ncbi.nlm.nih.gov/pubmed/20569214) [39] Correlations of method efficiency and viral load were performed using Spearman's rank test. Nonparametric statistics were employed due to the small number of *env* sequences that were amplified from the DBS and plasma of some subjects. Statistical significance was defined for *P* values of < 0.05.

**Nucleotide sequence accession numbers.** All SGA sequences determined in this study were deposited in GenBank under accession numbers KU901727–KU901998. Deep sequencing data are available in the NCBI sequence read archive under the accession number SRA307377.

## RESULTS

**Performance characteristics of the viral RNA extraction and SGA of HIV-1 from DBS.** To begin, we examined our capacity to isolate intact HIV-1 RNA for endpoint dilution PCR amplification of gp160 HIV-1 *env* from DBS stored at ambient temperatures for up to 3 weeks (21 days). We studied samples from 9 HIV-1-infected subjects (Table 1) and determined the number of gp160 sequences that could be amplified by standard SGA after TRIzol-based extraction (see methods) from a single DBS. This method was chosen based on DBS card recommendations, to isolate RNA preferentially over proviral DNA, to improve yields with a non-column-based isolation method, and to avoid unknown reagents in the final elution buffer that might affect subsequent PCR reactions [40]. We estimated individual blood spots contained between ~50 and 561,250 copies of HIV-1 RNA. This calculation was based on plasma viral load measurements for the 9 samples, which ranged from 1,710 to 22,450,000 copies/ml, the 50 μl quantity of blood per DBS, and the assumption that approximately 50% of whole blood volume is plasma. Extracted RNA underwent cDNA synthesis and endpoint dilution PCR and yielded reasonable numbers of gp160 *env* genes from each DBS (range 4-21 *env* sequences, median 10 per DBS) (Table 1). Notably, even DBS from subjects with the lowest viral loads (1,170 for F6817 and 2,200 for J6161) produced multiple amplicons per single DBS (Table 1). This number of gp160 *env* sequences from a single DBS suggests that the 5 DBS contained on commercially available cards could yield between 20 and > 100 sequences.

**Table 1.** Study subjects characteristics and number of SGA-derived *env* sequences. ^a.^ Subject was previously on ART, but had been of ART for > 3 months prior to 12/28/11 sample.

Subject	HIV status	Viral load (copies/ml)	Sample date	Antiretroviral treatment	DBS-derived *env* sequences	Plasma-derived *env* sequences
A8110	Chronic	1,464,000	12/28/11	Naive	16	14
K8072	Chronic	156,000	02/15/12	Naive	16	8
T4590	Chronic	155,000	12/15/11	Naive	10	15
T8107	Chronic	26,300	02/15/12	Naive	8	11
J7180	Chronic	15,800	01/18/12	Naive	9	10
J6161	Chronic	2,200	01/04/12	Naive	9	6
F6817	Chronic	1,710	12/28/11	Experienced^a^	4	12
T8250	Acute	13,400,000	04/24/12	Naive	21	21
N8261	Acute	22,450,000	05/17/12	Naive	11	12

Notably, there was no correlation between the viral load of the sample and the efficiency of SGA from DBS, as determined by Spearman correlation test (r: −0.220, *P*> 0.05). Indeed, similar *env* amplification efficiencies were observed for DBS RNA extracted with TRIzol-alone from subjects T8250 (14.1%), T4590 (11.8%), T8107 (11.2%), and F6817 (8.3%) despite their broad range of viral loads (range: 1,710–13,400,000 copies/ml) (Table 2).

**Table 2.** Effect of DBS storage time and temperature and extraction method on SGA efficiency.

Sample ID	Days DBS were kept at ambient temperature	Estimated HIV-1 copies per DBS^a^	Plasma TRIzol efficiency (%)	% efficiencies in DBS after short-term storage at ambient temperature		Subsequent transfer of DBS to -20°C for long term storage	
				DBS TRIzo-lalone extraction	DBS wa-ter-TRIzol extraction	Storage time at -20°C (months)	
J6161	1	55	66.7	41.0	26.7		
K8072	6	3,900	52.8	33.0	4.6	3.4	88.9
T8107	6	658	53.6	11.2	5.4	3.4	40.0
F6817	7	43	22.7	8.3	0.0	5.0	83.3
A8110	7	36,600	56.3	3.3	2.0	5.0	25.0
J7180	7	395	14.6	5.8	8.1		
T8250	7	335,000	18.8	14.1	0.9		
N8261	18	561,250	0.2	1.0	0.1		
T4590	20	3,875	88.5	11.8	8.2		

Median	7	3,875	52.80	11.20	4.60	3.0	19.4

Upon determining that we could amplify multiple intact gp160 env sequences, we next tested the efficiency of vRNA extraction from DBS compared with standard plasma viral RNA extraction methods. We compared DBS RNA extraction protocol to our gold standard procedure to extract vRNA from frozen plasma by automated commercial kit (QIAGEN QIAamp viral RNA mini kit). Of note, when tested in 9 plasma specimens, vRNA extraction by the TRIzol reagent protocol was substantially less efficient than the Qiagen protocol; approximately half the number (median 52.8%) of plasma SGA-derived *env* sequences were obtained by TRIzol compared to the QIAGEN protocol. We next compared TRIzol extraction from DBS to the QIAGEN protocol's automated extraction from plasma, which yielded a median of 11.2% as many sequences by volume (range 1–41.0%). Alternate extraction protocols, including using water coupled with TRIzol RNA isolation reagents, were less efficient, with a median of 4.6% (range 0.1–26.7%) (Table 2).

RNA integrity is a critical factor in PCR amplification efficiency, and HIV-1 RNA spotted on filter paper is well documented to be prone to degradation, but the rate of degradation reported has varied by study, environmental conditions, and desired application [21]. We evaluated SGA yields from DBS stored for different lengths of time at ambient temperature and −20°C. As seen in Table 2, short-term storage of DBS from 1 to 20 days at ambient temperature supported the generation of multiple SGA *env* amplicons. As predicted, SGA yields decreased with increased storage at ambient temperatures. Spearman rank correlation analysis showed a significant inverse correlation between DBS storage time and assay efficiency (r = −0.0703, *P* = 0.027). As shown in Table 2, DBS stored for either 1 or 6 days at room temperature had the highest *env* amplification efficiencies (see J6161 and K8072), whereas DBS stored for 18 days (N8261) had lesser amplification efficiencies. Further, a DBS stored for 24 days at room temperature failed to generate any full-length gp160 *env* amplicons despite a viral load of 29,920 copies/ml in an acutely infected subject, though amplification of multiple short fragments (~400 bp) was possible (data not shown).

Finally, we tested the capacity to preserve vRNA integrity when DBS were promptly (*within 1-20 days*) frozen. For five subjects, DBS were stored at −20°C for 3-5 months (Table 2). In each of these samples, viral RNA integrity was largely preserved, allowing successful amplification of full-length *env* genes with only modestly reduced efficiency (19–89%) when compared to baseline DBS (Table 2).

**Figure 1.**
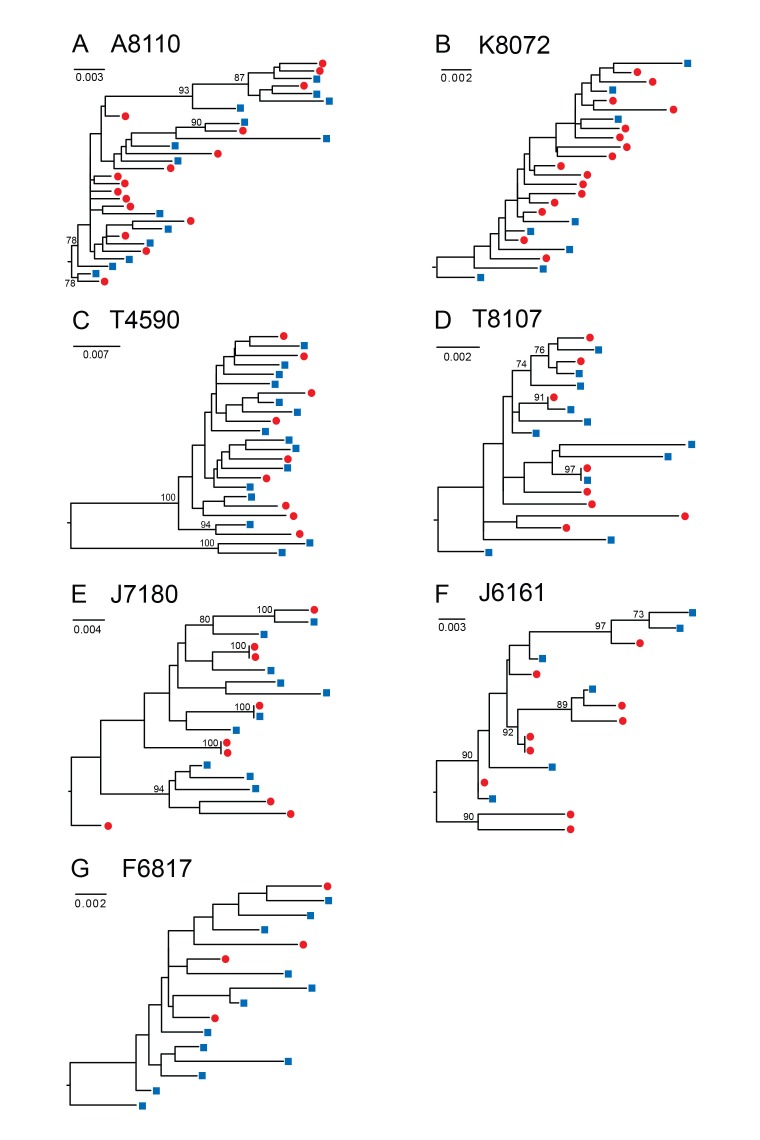
**HIV-1 *env* sequences derived from DBS are genetically heterogeneous and phylogenetically indistinguishable from plasma variants**. Maximum likelihood phylogenetic analyses are shown for gp160 HIV-1 *env* nucleotide sequences amplified by SGA from DBS (red circles) and plasma (blue squares) from seven chronically HIV-infected subjects (A to G). A single *env* sequence from subject T4590 (C) was excluded due to a large deletion. Numerals at nodes indicate approximate likelihood ratio test values of ≥ 0.73. The scale bar represents the number of nucleotide substitutions per site.

**Supplemental Figure 1.**
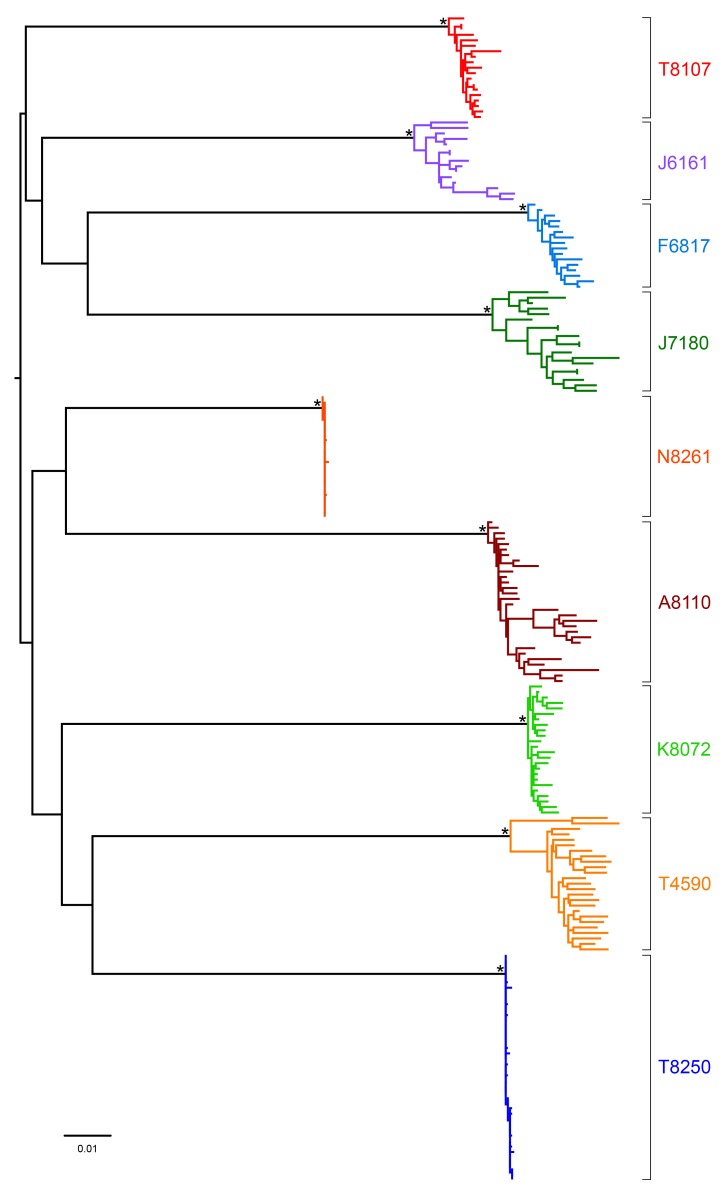
Maximum-likelihood phylogenetic tree of all study participants. The 9 study subjects' SGA-derived gp160 *env* sequences cluster independently, demonstrating a lack of cross-contamination. Sequences are color-coded by subject. Genetic distance is indicated by the scale bar.

SGA-derived HIV-1 *env* sequences from chronically infected subjects are phylogenetically similar in paired DBS and plasma samples. To determine the capacity of DBS to replace plasma as a sample platform for SGA, we compared the gp160 *env* sequences generated from each sample source in a panel of HIV-1 infected subjects. We amplified 228 full-length HIV-1 *env* genes (range 15-42, median 23 sequences per subject) from paired plasma and DBS samples obtained from 7 subjects with chronic HIV-1 infection who were off antiretroviral therapy (ART) (Fiebig stage VI; n = 6 antiretroviral treatment naïve subjects and n = 1 subject with previous ART experience) (Table 1). The subjects had plasma viral loads ranging from 1,710 to 22,450,000 copies/ml, allowing us to test a broad range of input HIV-1 viral loads. We performed RNA extraction, cDNA synthesis and SGA from the DBS and plasma samples and compared the SGA-derived sequences phylogenetically (Figure 1). For these chronically infected subjects, maximum likelihood phylogenetic analyses showed that DBS *env* sequences were phylogenetically heterogeneous and indistinguishable from those derived from plasma; that is, DBS *env* sequences were interspersed among plasma *env* sequences in phylogenetic trees (Figure 1). As has been previously reported in chronic HIV-1 quasispecies, clusters of identical or near-identical sequences were noted in three chronically infected subjects (Figure 1D, E, and F), suggesting recent clonal expansion of some variants [41, 42]. In two subjects (Figure 1 D, E), these identical sequences were derived from DBS and plasma sources, reinforcing the idea that comparable populations were sequenced by the different methods. To test for bias associated with DBS vs plasma source, we tested for differences in viral diversity and compartmentalization (Table S1). Intra-patient *env* sequence diversity was not significantly different in any individual when tested by a two-sample test for sequence diversity. Using a phylogenetically-based Slatkin-Maddison test to investigate whether viruses from DBS had any evidence of compartmentalization, we compared the minimum possible number of intercompart-mental migration events to the frequency distribution of migration events in 1,000 randomized trees [35], and found that none of the 7 chronically HIV-infected and 2 acutely infected subjects examined showed statistical evidence of compartmentalization in DBS. Last, phylogenetic analysis of all *env* sequences demonstrated no evidence of cross-contamination between samples, as each subject's sequences clustered in separate branches of the tree with inter-strain diversities typically observed for subtype B viruses (Figure S1).

**Figure 2.**
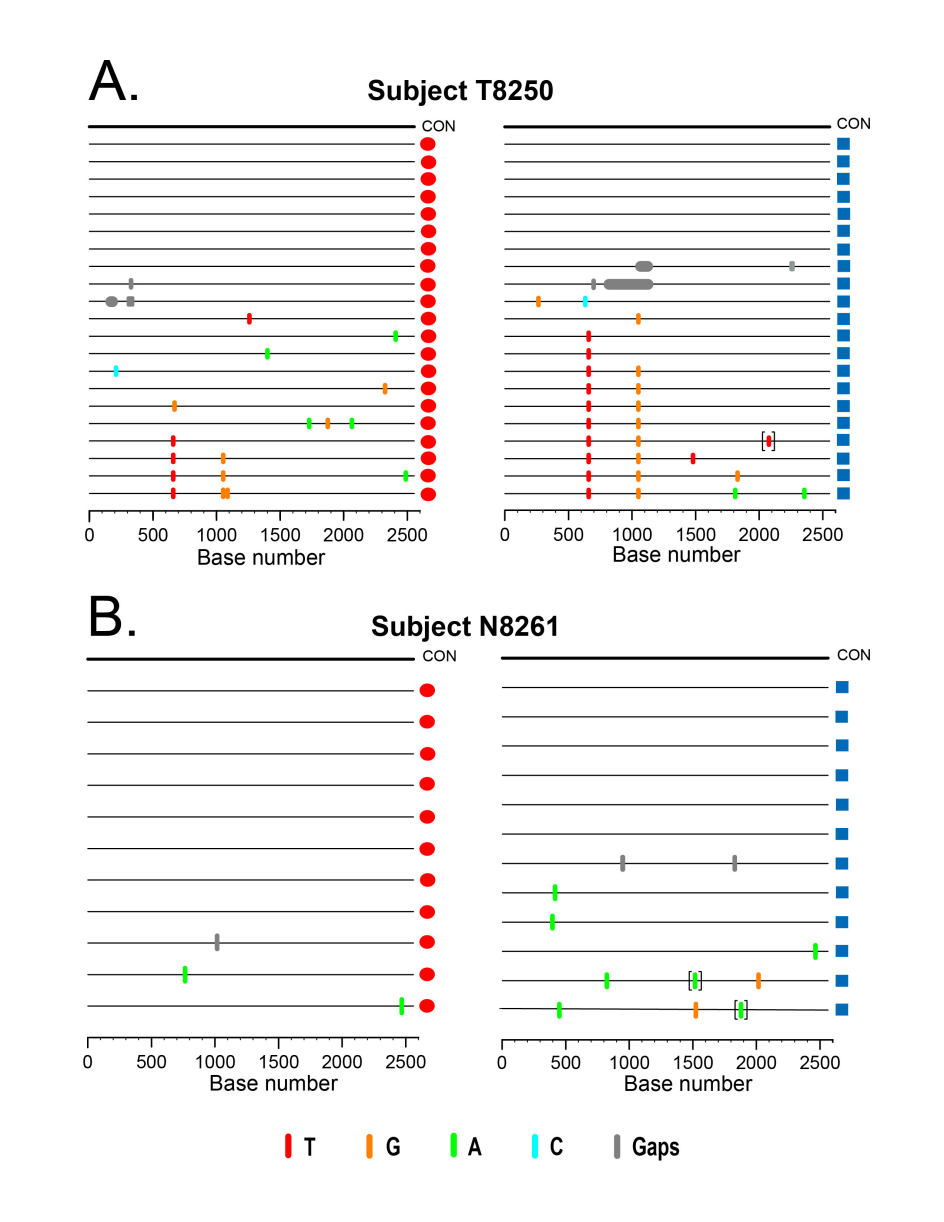
**HIV-1 *env* sequences from paired DBS-plasma samples coalesce to a common ancestor in two acutely infected subjects.** Highlighter plots of gp160 *env* sequences derived from DBS (red circles) or plasma (blue squares) from subjects T8250 (A) and N8261 (B) exhibit low genetic diversity and coalesce to identical TF *env* sequences for each subject. In T8250, shared polymorphisms occur at the same two nucleotide positions in both DBS and plasma sequences. Nucleotide differences from the inferred consensus sequence are indicated by tic marks color-coded for each base or gaps due to nucleotide deletions; tics inside brackets indicate nucleotide insertions. The horizontal axis indicates HXB2 *env* nucleotide positions.

**Identification of a single HIV-1 TF gp160 *env* gene using either DBS or plasma.** We next examined the feasibility of using DBS as an alternative sample matrix to infer the TF HIV-1 *env* gene sequence. Figure 2 depicts Highlighter plots of the SGA-derived gp160 *env* sequences from DBS and plasma from 2 acutely infected subjects. In Figure 2A and B, the sequences from subject T8250 demonstrate similar low diversity lineages for DBS and plasma-derived sequences, which coalesce into a single, identical consensus sequence that represents the TF *env.* The DBS and plasma sequences each demonstrate random mutations as well as two sites of shared polymorphisms (a synonymous mutation at HXB2 position 6881 and a non-synonymous mutation at position 7279), which likely represent early stochastic mutations or selection. Importantly, they are at identical positions in the DBS and plasma derived sequences. Sequences from Subject N8261 (Figure 2C, D) also demonstrate low-diversity lineages with largely identical sequences that coalesce to a single, shared TF *env* sequence for both DBS and plasma sequence sets. Thus, for both subjects, a low-diversity lineage coalesces to an unambiguous consensus sequence that is identical for DBS and plasma sequences. Importantly, as predicted for all TF viruses [4], the deduced amino acid sequence of the inferred consensus *env* gene corresponding to subjects T8250 and N8261 had intact open reading frames for the *env* gene and partial sequences coding for *tat*, *rev*, and *vpu* genes. Of interest, in both subjects the DBS sequences had a higher proportion of identical sequences (38% vs 29% and 73% vs 58%, for T8250 and N8261, respectively), a trend towards lower estimated time to MRCA (27 vs 28 days for T825 and 6 vs 19 for N8261, with overlapping 95% CI), and better conformed to a model of random virus replication in acute HIV-1 infection when compared to plasma sequences (Table 3) [4, 43]. Strong inter-assay reproducibility was observed in the SGA sequences generated for the two acutely infected individuals. For both T8250 and N8261, SGA was performed in multiple independent sessions, each of which yielded the identical amino acid TF sequence.

**Table 3.** Statistics and mathematical model estimates of the most recent common ancestor (MRCA) of HIV *env* sequences derived from DBS and plasma in two acutely infected individuals

Sample ID	Total number of sequences	Maximum length of sequence	Maximum HD	Mean HD (%)	Poisson estimated days since MRCA Lamda (95% CI)	Lamda	Standard Deviation	Goodness of ft *P*-value	HD fit to Poisson	Star phylog-eny
T8250 DBS	21	2556	6	1.60	27 (17, 37)	1.624	0.293	0.246	Yes	Yes
T8250 plasma	19	2557	6	1.70	28 (20, 35)	4.684	0.229	0.307	Yes	No
N8261	11	2559	2	0.36	6 (0, 12)	0.364	0.177	0.656	Yes	Yes
N8261 plasma	12	2560	4	1.20	19 (9, 30)	1.167	0.316	0.856	Yes	Yes

**Figure 3.**
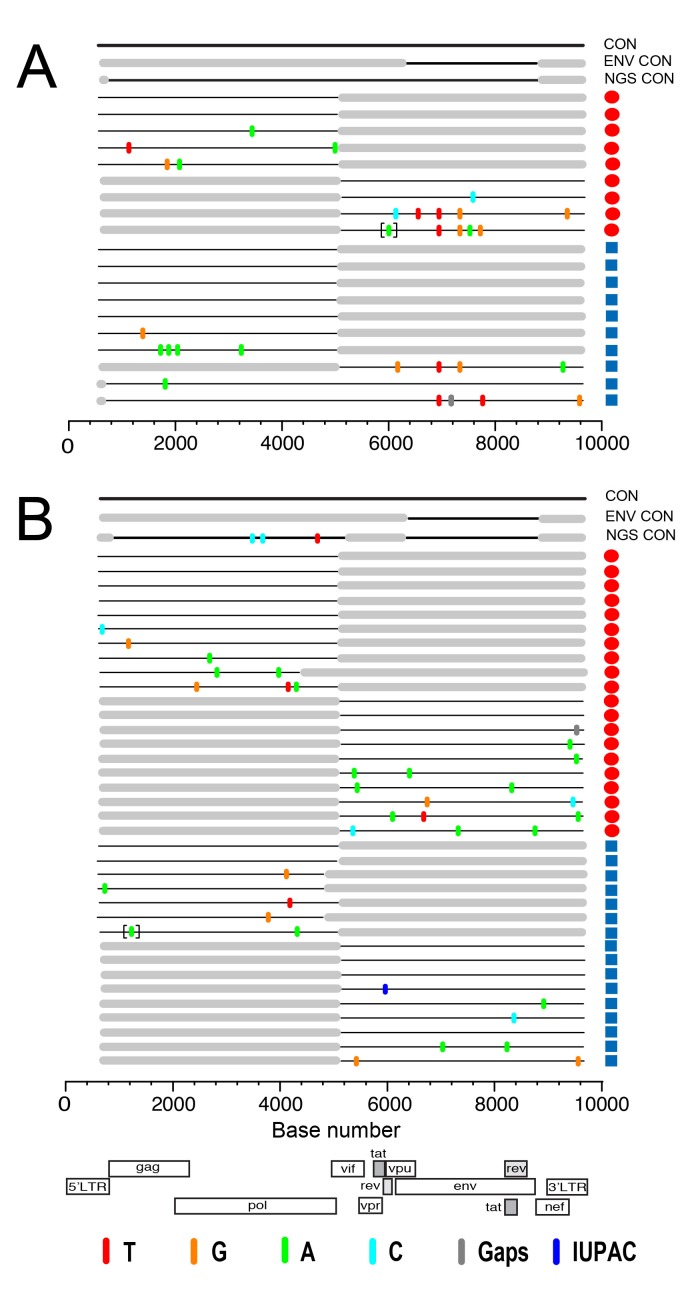
**Comparison of whole HIV-1 genomes from DBS by SGA and deep sequencing in two acutely infected subjects.** Half- and full-genome HIV-1 sequences derived from DBS (red circles) and plasma (blue squares) from subjects T8250 (A) and N8261 (B) are aligned to a patient-specific consensus master sequence from SGA sequences (top line), gp160 *env* sequences (second line from top), and 454 deep sequences (third line). Colored tics and brackets are as defined in Figure 2. Flanking gray boxes indicate regions not amplified, while gray tics indicate intergenomic deletions. The horizontal axis indicates nucleotide positions based on HXB2 reference sequence numbering. The HIV-1 genome cartoon at the bottom depicts the flanking LTRs and 9 canonical protein-coding genes.

**Identification of the full-length HIV TF genome by SGA and next generation sequencing from DBS.** We aimed to infer the complete nucleotide sequence of the TF HIV-1 virus in paired DBS-plasma samples. SGA analysis of 5′-half (n = 12), 3′-half (n = 5), and near-full length 9 Kb (n = 2) genomes generated from DBS or plasma coupled with the larger set of gp160 *env* sequences allowed the identification of the full-length TF HIV-1 genome in subject T8250 (Figure 3A). Half-genome sequences showed a Poisson distribution of mutations coalescing to a single consensus. Shared polymorphisms seen in the *env* gene region were resolved by using the larger set of *env*-only sequences; there were no additional shared polymorphisms outside the *env* gene. 5′-half genome sequences were highly homogeneous and had no ambiguous positions, thus allowing the identification of the full-length TF genome of subject T8250. Highlighter analysis of 5′-half (n = 17) and 3′-half (n = 19) HIV-1 genome sequences from subject N8261 also revealed a Poisson distribution of mutations (Figure 3B) which, together with the gp160 *env* sequences, allowed inference of the identical single transmitted/founder HIV-1 genome using sequences from both DBS and plasma.

**Figure 4.**
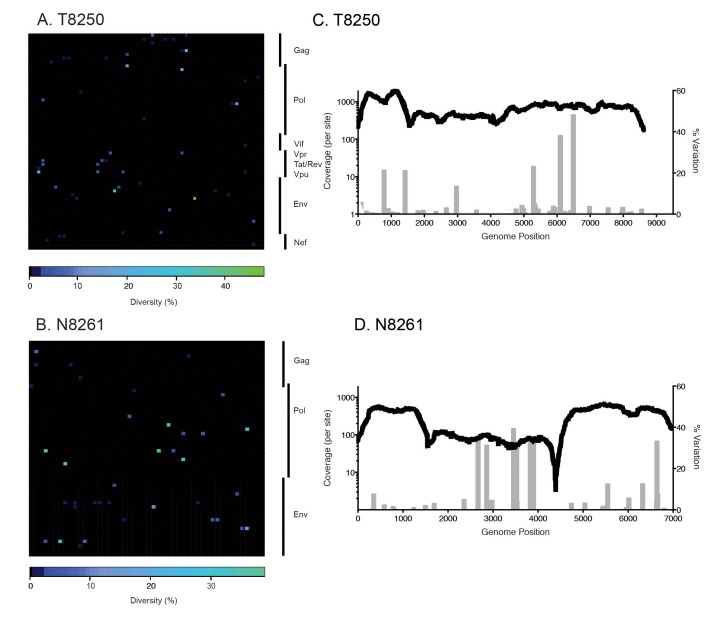
**454 deep sequencing of TF HIV-1 progeny in two subjects with acute infection.** Heat maps illustrate both non-synonymous and synonymous codon sequence diversity of HIV-1 from DBS RNA in subjects T8250 (A) and N8261 (B). The plots are a graphical representation of the frequency of the intra-host sequence diversity with respect to the dominant codon residue (% diversity) for each codon (x-axis) of individual canonical HIV-1 proteins (y-axis). Each square represent a single codon, colored to reflect the percentage of reads that differ from the consensus residue. The diversity scale is color-coded with completely conserved residues in black and marine-blue tones for low-frequency polymorphisms in codons (< 10%), sky-blue tones for those with moderately high frequency (10–30%) and green for high frequency polymorphisms (> 30%). The entire HIV-1 proteome of subject T8250 is represented in the upper plot, whereas the *pol*, *gag*, and *env* proteins are depicted for patient N8261 in the lower plot. Coverage plots depicting the coverage (sequencing reads per site, black line) and the level of variation (gray bars) at a given codon across the sequenced region of the genome are shown for subjects T8250 (C) and N8261 (D).

We next assessed the capacity for DBS samples to be used in a next-generation sequencing (NGS) approach to identify the TF genome. With greater sequencing depth than standard SGA, NGS has improved sensitivity to detect minor variants (see sensitivity analysis in [4]). A direct comparison of a T8250 HIV-1 consensus sequence built from NGS sequencing reads (HXB2 positions: 501– 9606) showed a 100% match with the consensus inferred by SGA of DBS and plasma (Figure 3A). Nucleotide polymorphisms are shown in heat map format in Figure 4, and demonstrate that the vast majority of codon sites exhibited low diversity, with the minor variant generally comprising less than 10% of reads. In particular, the two shared polymorphisms in the *env* gene detected by SGA were also observed by NGS at modestly higher frequencies.

The GCC to GCT synonymous mutation at HXB2 position 6881 occurred in 38% of 336 high quality deep sequencing reads and 19% (4 out of 21) of SGA-derived *env* sequences from DBS, while the CAA to CGA non-synonymous mutation at position 7279 occurred in 48% of 509 deep sequencing reads (shown in green on the heatmap in Figure 4A) and 14% (3 out of 21) of SGA-derived *env* sequences. A third shared polymorphism detected in 23% of deep sequencing reads (547 HQ read depth) in the *vpu* gene (HXB2 position 6072) was observed in 25% (1 out of 4) of 3′-half sequences from DBS. Four additional shared polymorphisms that were not sampled by SGA were detected by NGS at frequencies ranging from 6% to 21% in the *gag*/*pol* region (HQ read depth range: 164–712) (Figure 4C). In N8261, the consensus sequence constructed from NGS sequencing reads showed a 99.96% match with the consensus sequence inferred by SGA of DBS and plasma (Figure 3B) with only 3 nucleotide differences in *pol* distinguishing the 2 methods. For the 3 nucleotide discrepancies, the number of NGS quality reads was relatively low (< 100) (Figure 4D) and the SGA consensus bases were observed, but represented minor variants (frequency of 31–35%). Finally, as predicted, the inferred TF genome of the virus in both T8250 and N8261 coded for an intact viral proteome for all major canonical HIV-1 proteins.

## DISCUSSION

In the present study, we tested the feasibility of using DBS to amplify full-length HIV-1 *env* genes by SGA and deep sequencing to assess sequence diversity in chronic infection and identify the full-length TF HIV-1 genome (or *env* gene) in recently infected individuals. We found that SGA from vRNA extracted from matched DBS and plasma were phylogenetically indistinguishable in representing the diversity of chronic HIV-1 quasispecies. This demonstration suggests that sophisticated sequence analyses are possible in research subjects and clinical trial participants throughout the world, including those sampled in locations far from a centralized laboratory facility. We also found that sequences from matched DBS and plasma samples identified a single TF virus with an identical *env* gene and full-length HIV-1 genome sequences by SGA and deep sequencing methodologies. Our study provides proof of concept that DBS can be used for both SGA and deep sequencing methods that form the cornerstone of modern molecular virology research.

While less efficient than sequencing vRNA extracted from plasma that is flash frozen and stored at −80°C, vRNA yields from DBS are sufficient for the vast majority of viral analyses currently employed, including identification of the complete TF genome. For *env* analyses, each DBS yielded between 4 and > 20 gp160 *env* sequences. Given the 5 DBS per typical commercially available DBS card and the potential to relatively easily collect multiple cards per subject at one time, the 10–30 sequences generally used to infer the TF virus [1–5, 14, 15], are achievable. Similar numbers of sequences would be adequate to measure other viral parameters, including the characterization of the complex viral populations that could break through a suppressive and/or curative therapy and characterization of the HIV-1 quasispecies in an actively viremic individual off antiretroviral therapy.

Our analysis found that the efficiency of SGA from DBS was dependent on several factors, including the RNA extraction method and storage time and temperature. We utilized a fairly standard vRNA isolation procedure to achieve sufficient yields to perform SGA; expectedly, variations on this protocol altered the efficiency of this process. As demonstrated in a recent study by Monleau and colleagues [40], additional RNA isolation assay optimization could increase efficiency further. As expected, we found that nucleic acid degradation occurred with DBS stored for weeks at ambient temperatures and, to a lesser extent, for months at −20°C. The fact that storage of a single DBS sample for just 1 day at ambient temperature showed the highest efficiency in *env* amplification (41% using a TRIzol-alone protocol) compared to the significant lower efficiencies after 6–20 days of storage (Table 2) suggest that either prompt processing or immediate transfer of DBS cards to −20°C should result in higher PCR yields. The latter is supported by the observation that DBS placed at −20°C within three weeks of collection preserved 19.4% to 88.9% of the RNA molecules remaining intact after the initial storage at ambient temperature, and those placed in freezers earlier (within a week) were even better preserved (Table 2). Further, DBS cards stored for months in non-frost-free −20°C freezers maintained much of their viability. Though we do not expect RNA degradation to be biased towards any particular RNA populations and do not suspect it would affect phylogenetic patterns, we did not directly test this. The World Health Organization recommends ≤ 14 days at ambient temperature after collection of DBS, which are then stored at −20°C or −80°C for monitoring HIV-1 drug resistance [28, 44]; this protocol would similarly maintain viability for SGA. A limitation of our study was the lack of assessment of the effects of the warm, humid conditions experienced in many tropical field settings. While this has decreased the efficiency of DBS-based assays in other studies [27, 45–47], we note the recent report from Zambia demonstrating successful PCR amplification of the HIV-1 DNA template from DBS [29]. While Seu and colleagues did not report sequencing results, their results strongly suggest that PCR and SGA are feasible in resource-limited field settings, though additional, larger field studies are warranted.

Several findings from the comparisons of matched DBS and plasma-derived acute HIV sequences suggest that our DBS sequences represent a mixture of plasma HIV-1 RNA and cell-associated proviral DNA (or cell associated RNA). To begin, we found that plasma viral load measurements did not correlate with the efficiency of SGA. Indeed, samples with orders of magnitude differences in plasma viral load had relatively similar efficiencies in gp160 *env* amplification (Table 2). Further, compared to acute plasma-derived sequences, matched DBS sequences showed a trend towards greater identity with the TF virus and lower estimates of time to MRCA (Table 3). Cell-free HIV-1 plasma virions have remarkably short half-lives (< 1 day) [48] and represent only the most recent rounds of viral replication. Cellular proviral DNA or cellular RNA, in contrast, may represent longer-lived virus sequences from replication cycles occurring days to weeks earlier. These more archival sequences therefore lack the additional random polymorphisms that accrue over time and are genetically closer to the TF virus. The discordance between plasma viral load and SGA sequence yield coupled with more ancestral (more TF-like virus) populations sampled by DBS suggest that DBS contain both plasma vRNA and proviral DNA. While we used a nucleic acid isolation protocol designed to preferentially extract RNA over DNA, it is likely that a certain amount of proviral DNA was also isolated. The presence of proviral DNA in DBS is well established [22, 28]; a recent study by Masciotra and colleagues employing nonspecific nucleic acid extraction techniques found that as many as half of tested samples had amplifiable proviral DNA, with greater frequencies (up to 58%) in samples with higher plasma viral loads [22]. In addition, storage at ambient temperature may lead to degradation of the less stable vRNA and relative overrepresentation of DNA [27, 45–47]. Interestingly, increased representation of HIV DNA may actually be advantageous for TF identification. SGA of plasma vRNA and proviral DNA have been validated as equally able to identify the TF virus in acute samples [5] and the ability to amplify sequences representing earlier viral populations is beneficial to TF virus inference. In clinical specimens with low viral loads in chronically infected individuals, whose proviral DNA may reflect archived or defective virus, DNase treatment of DBS extracts should be considered to better reflect actively replicating plasma viral RNA [47].

As expected in the complex chronic HIV quasispecies, which are substantially more diverse and responding to multiple selective immune responses, subtle differences in timing between free plasma virus and cell-associated virus were not detected. Phylogenetically, paired DBS-plasma samples were highly similar; in fact, identical sequences from DBS and plasma were identified in several patients (Figure 1) and suggest that DBS and plasma are relatively equivalent specimens with which to characterize the circulating chronic HIV-1 viral quasispecies. Thus, we conclude that RNA-targeting nucleic acid isolation from DBS results in vRNA and some fraction of proviral DNA or cell-associated RNA that are less represented in acellular plasma. Importantly, this DNA does not alter the capacity for DBS to be used to accurately identify the TF virus and/or characterize chronic viral quasispecies. This conclusion echoes similar findings from previous studies of DBS-based assessments of clinically relevant HIV parameters, including plasma viral load and genotypic drug resistance, wherein subtle differences between matched DBS and plasma assays were attributed to contributions from DNA, but results from the matched samples were largely equivalent (reviewed in [21]).

In summary, this study demonstrates the feasibility of DBS as specimens for TF identification and viral diversity analyses in general. DBS provides significant cost savings and logistical advantages over standard plasma specimens. The capacity to utilize DBS in technically demanding molecular virology techniques expands opportunities for comprehensive and sophisticated studies in clinical trials and translational research to resource-limited settings across the world, including places where HIV-1 is most prevalent and intervention is most critical.

## SUPPLEMENTARY MATERIALS

**Supplementary Table 1.** HIV-1 env diversity and compartmentalization tests between two viral populations (plasma vs DBS) a) Rank-ordered diversity data were divided into four equal parts using quartiles: first (Q1), third (Q3) quartiles and interquartile range (IQR = Q3-Q1) are shown as a measure of variability. b) S denotes the number of changes (in parsimony steps) of a character corresponding to population membership.c) Probabilty of S or fewer steps in 1,000 randomized tree assignments.

	Group	Number of sequences	Intra-patient *env* sequence diversity				Two-sample test for sequence diversityy				Slatkin-Maddison Testy	
			Median	Q1 ^a^	Q3 ^a^	IQR ^a^	T score:	Degrees of freedom:	Z-test *P* value:	T-test *P* value:	N-steps (S) ^b^	*P*-value ^c^
A8110	plasma DBS	14 16	1.478 1.078	0.887 0.590	1.913 1.480	1.026 0.890	−1.588	33.23	0.112	0.122	12	0.968
K8072	plasma DBS	8 16	0.611 0.547	0.504 0.462	0.759 0.654	0.256 0.191	−0.715	93.40	0.475	0.477	6	1
T4590	plasma DBS	15 10	1.271 1.147	1.102 1.059	2.175 1.236	1.073 0.177	−1.669	2110.21	0.095	0.095	9	1
T8107	plasma DBS	11 8	0.590 0.548	0.462 0.482	0.675 0.674	0.213 0.192	0.039	7.02	0.969	0.970	7	0.799
J7180	plasma DBS	10 9	1.874 1.484	1.314 1.145	2.067 2.150	0.753 1.005	−0.581	0.84	0.561	0.680	6	0.432
J6161	plasma DBS	6 9	1.189 1.316	0.932 0.826	1.402 1.661	0.470 0.835	0.294	0.58	0.768	0.841	5	0.712
F6817	plasma DBS	12 4	0.715 0.716	0.589 0.631	0.842 0.759	0.254 0.129	−0.076	2.61	0.940	0.945	4	1
T8250	plasma DBS	21 21	0.050 0.042	0.042 0.042	0.084 0.126	0.042 0.084	0.523	19.71	0.601	0.607	13	0.321
N8261	plasma DBS	12 11	0.000 0.000	0.000 0.000	0.042 0.042	0.042 0.042	−0.314	52.45	0.753	0.755	8	0.645
